# Prognostic analysis of anoikis-related genes in bladder cancer: An observational study

**DOI:** 10.1097/MD.0000000000038999

**Published:** 2024-07-19

**Authors:** Fu Huang, Liquan Zhou, Junjie Sun, Xihua Ma, Yongfeng Pei, Qiuwen Zhang, Yanqing Yu, Guining He, Lirong Zhu, Haibin Li, Xiaoming Wang, Fuzhi Long, Haipeng Huang, Jiange Zhang, Xuyong Sun

**Affiliations:** a Institute of Transplantation Medicine, The Second Affiliated Hospital of Guangxi Medical University; Guangxi Clinical Research Center for Organ Transplantation; Guangxi Key Laboratory of Organ Donation and Transplantation, Nanning, PR China; b Department of Urology, The Second Affiliated Hospital of Guangxi Medical University, Nanning, PR China

**Keywords:** anoikis, bladder cancer, chemotherapy drug sensitivity, risk model, risk score

## Abstract

Anoikis is proved to play a crucial role in the development of cancers. However, the impact of anoikis on the prognosis of bladder cancer (BLCA) is currently unknown. Thus, this study aimed to find potential effect of anoikis in BLCA. The Cancer Genome Atlas (TCGA)-BLCA and GSE13507 cohorts were downloaded from TCGA and the Gene Expression Omnibus (GEO) databases, respectively. Differentially expressed genes (DEGs) were screened between BLCA and normal groups, which intersected with anoikis-related genes to yield anoikis-related DEGs (AR DEGs). Univariate COX, rbsurv, and multivariate COX analyses were adopted in order to build a prognostic risk model. The differences of risk score in the different clinical subgroups and the relevance between survival rate and clinical characteristics were explored as well. Finally, chemotherapy drug sensitivity in different risk groups was analyzed. In total, 78 AR DEGs were acquired and a prognostic signature was build based on the 6 characteristic genes (CALR, FASN, CSPG4, HGF, INHBB, SATB1), where the patients of low-risk group had longer survival time. The survival rate of BLCA patients was significantly differential in different groups of age, stage, smoking history, pathologic-T, and pathologic-N. The IC_50_ of 56 drugs showed significant differences between 2 risk groups, such as imatinib, docetaxel, and dasatinib. At last, the results of real time quantitative-polymerase chain reaction (RT-qPCR) demonstrated that the expression trend of CALR, HGF, and INHBB was consistent with the result obtained previously based on public databases. Taken together, this study identified 6 anoikis-related characteristic genes (CALR, FASN, CSPG4, HGF, INHBB, SATB1) for the prognosis of BLCA patients, providing a scientific reference for further research on BLCA.

## 1. Introduction

Bladder cancer (BLCA) is a malignant tumor that originates from the epithelial tissue of the bladder’s urinary tract, and the incidence of BLCA is higher in male patients than in female patients.^[1]^ BLCA is 1 of the common cancers, with the highest incidence rates in Europe and North America.^[2]^ In 2022, the number of new BLCA is expected to be 81,180, and the number of deaths medical records is 17,100.^[3]^ Currently, treatment methods for BLCA include surgery, chemotherapy, and radiotherapy, among others. For early-stage BLCA patients, surgical resection has good efficacy.^[4]^ For advanced BLCA patients, combination therapy involving surgical resection, radiotherapy, and chemotherapy is used, but the cure rate is low.^[5]^ Despite advances in diagnosis and treatment, the prognosis for advanced BLCA remains poor. Therefore, the identification of new biomarkers related to BLCA patients’ survival is crucial for improving prognosis and developing personalized treatment strategies.

Anoikis is a programmed cell death mechanism, and occurs in cells that have lost their attachment to the extracellular matrix.^[6]^ Several specific factors, including growth proteins, pH levels, transcriptional signaling pathways, and oxidative stress, have been identified as the drivers of anoikis resistance (AR), leading to increased cancer proliferation and metastasis.^[7]^ Resistance to anoikis leads to tumor cell survival and metastasis in gastric^[8]^ and prostate cancer.^[9]^ Chen et al identified anoikis-related genes (ARGs) features (CHEK2, PDK4, ZNF304, SNAI2, SRC) that can predict the prognosis of renal clear cell carcinoma patients, and demonstrated that the risk model is associated with the immunosuppressive microenvironment.^[10]^ Zhang et al developed a risk model for predicting the prognosis of BLCA patients based on 7 anoikis-related long noncoding RNAs (lncRNAs) (LINC01767, AC011503.2, UBE2Q1-AS1, Z99127.1, AC112721.2, MAFG-DT, LINC00456) that are associated with apoptosis. Moreover, the anoikis-related lncRNAs may help to classify BLCA patients and assess their immune status. However, there is still limited research on the correlation between BLCA and anoikis until now.^[11]^

Although it has been proven that anoikis is associated with the progression and metastasis of various tumors, the relationship between BLCA and ARGs is still unclear. We obtained BLCA-related datasets from The Cancer Genome Atlas (TCGA) and the Gene Expression Omnibus (GEO) databases, and identified characteristic genes that are associated with the survival of BLCA patients, and explored the correlation between these genes and clinical features as well as drug sensitivity in BLCA. This study could provide potential targets for clinical diagnosis and a theoretical basis for further interpretation of BLCA.

## 2. Materials and methods

### 2.1. Data sources

The TCGA-BLCA cohort was downloaded from TCGA database (https://portal.gdc.cancer.gov), containing 400 BLCA and 18 normal tissue samples. These samples all had survival information. BLCA-related dataset GSE13507 (GPL6102), containing 165 primary BLCA samples and 9 normal bladder mucosa samples was downloaded from GEO database (https://www.ncbi.nlm.nih.gov/geo/). In total, 362 ARGs were got via GeneCards database by searching keyword Anoikis (relevance score > 1) (https://www.genecards.org).

### 2.2. Identification of differentially expressed genes (DEGs) between BLCA and normal groups

Firstly, the differences of mRNA expression between BLCA and normal groups in TCGA-BLCA dataset were compared via “differential expression analysis” in GEPIA with |log_2_fold change (FC)| > 1 and *q*-value < 0.05. Differentially expressed genes (DEGs) 1 were then identified. Additionally, we also analyzed DEGs 2 between BLCA and normal groups in TCGA-BLCA dataset via “DEseq2” R package (version 1.36.0) with |log_2_FC| > 1 and adj *P* < .05.^[12]^ Finally, key DEGs were obtained through taking the intersection of DEGs 1 and DEGs 2.

### 2.3. Characterization and enrichment analysis of anoikis-related DEGs (AR DEGs)

Venn tool was utilized to visualize the AE DEGs which were obtained by overlapping key DEGs and ARGs. Furthermore, we performed the Gene Ontology (GO) and Kyoto Encyclopedia of Genes and Genomes (KEGG) enrichment analyses of AR DEGs through “clusterProfiler” R package (version 4.4.4) (adj *P* < .05).

### 2.4. Establishment of the risk model and the creation of nomogram

Based on AR DEGs, univariate Cox analysis was firstly performed to screen BLCA survival related genes with hazard ratio (HR) ≠ 1, *P* < .05 through survival package (version 3.4.0).^[13]^ After obtaining BLCA survival related genes, rbsurv algorithm was processed to further screen candidate characteristic genes using rbsurv package (version 2.54.0).^[14]^ Triple cross-validation was used to randomly allocate the training set and validation set according to 2:1 in TCGA-BLCA, and the maximum number of genes was 30, the analysis was repeated for 1000 times. The optimal model was selected, and genes in this model were identified as candidate characteristic genes. Furthermore, the characteristic genes were finally selected through multivariate Cox analysis, when the model was optimal. The model was constructed with above obtained characteristic genes, and risk score of BLCA was calculated with following formula: $\text{Risk }\text{​}\text{​}\;\text{​}\text{​}\text{ score }\text{​}\text{​}\;\text{​}\text{​}\;=({\text{coef}}_{i}*{\text{X}}_{i})$. Each BLCA patient could obtain a score via formula while patients were classified into high- and low-risk groups via the median value of the score. We carried out Kaplan–Meier (K-M) survival curves and Receiver operating characteristic (ROC) analysis to explore the efficacy of risk model. The GSE13507 was utilized as an external validation set for the risk scoring model.

According to risk score and clinical features (age, gender, T/N stage, stage, smoking history), independent prognostic factors were screened by univariate and multivariate Cox analyses with criteria of HR ≠ 1 and *P* < .05. The nomogram was created with the goal of predicting the survival of BLCA patients at 1, 3 and 5 years. Furthermore, corresponding calibration curves and ROC curves were plotted to assess the reliability of nomogram.

### 2.5. Analysis of clinical correlation

The differences of risk score were analyzed between groups with clinical characteristics to assess the relevance between them. The clinical characteristics were age (≤60/>60), gender (female/male), stage (stage I/II/III/IV), pathologic-T (T1/2/3/4), pathologic-N (N0/1/2/3), and smoking history (1/2/3/4/5). Moreover, we plotted K-M curves for different stage of clinical characteristics to explore the relevance between clinical characteristics and survival rate of BLCA patients (*P* < .05).

### 2.6. Screening and enrichment analysis of DEGs between high- and low-risk groups

The DEGs 3 were screened between 2 risk groups via “DEseq2” R package with |log_2_FC| > 1 and adj *P *< .05. To analyze which pathways that DEGs 3 enriched, we performed the enrichment analysis for DEGs 3 between 2 risk groups via “clusterProfiler” R package (version 4.4.4) (adj *P* < .05).

### 2.7. Drug sensitivity analysis

To assess the sensitivity to disease conventional chemotherapeutic agents in 2 risk groups, the half maximal inhibitory concentration (IC_50_) of 138 chemotherapy drugs was calculated via the “pRRophetic” R package (version 0.5) for each BLCA patient,^[15]^ and the differences were compared by Wilcoxon text (*P* < .05). In addition, the correlation between risk score and IC_50_ of differential drugs was calculated via Spearman algorithm.

### 2.8. Expression analysis of characteristic genes

To further demonstrate the responsibility of our results, the expression levels of the characteristic genes was compared in BLCA and normal groups in the TCGA-BLCA cohort and GSE13507 dataset.

### 2.9. Sample collection

In our study, the 5 patients with BLCA at the second affiliated hospital of Guangxi Medical University from May 2022 to April 2023 were recruited. The 5 BLCA tissue samples and 5 normal adjacent tissue samples were gained. This study was approved by the Ethical Review Committee of the second affiliated hospital of Guangxi Medical University. All patients had signed an informed consent form.

### 2.10. Real time quantitative-polymerase chain reaction (RT-qPCR)

The expression of the 6 characteristic genes was further validated via RT-qPCR. The ten samples’ total RNA was extracted using TRIzol (Ambion, Austin, USA) in accordance with the instructions provided by the manufacturer. The SureScript-First-strand-cDNA-synthesis-kit (Servicebio, Wuhan, China) was utilized to perform reverse transcription of total RNA to cDNA the based on the manufacturer’s instructions. RT-qPCR was performed utilizing the 2xUniversal Blue SYBR Green qPCR Master Mix (Servicebio, Wuhan, China). The primer sequences for RT-qPCR were shown in Table 1. The internal reference gene was GAPDH. The 2^−ΔΔCt^ method was utilized to calculate the expression of diagnostic genes.

**Table 1:** The primers used for real time quantitative-polymerase chain reaction (RT–qPCR).

Gene name	Primer sequence (5′–3′)
INHBB-F	CGAAATCATCAGCTTCGCCG
INHBB-R	GCTTGAGGTCCACCCTCTTC
FASN-F	CCGAGACACTCGTGGGCTA
FASN-R	CTTCAGCAGGACATTGATGCC
SATB1-F	GCATCCTTTCCCGGTCCAT
SATB1-R	TGGACCCTTCGGATCACTCA
CALR-F	CTCTGGCAGGTCAAGTCTGG
CALR-R	CTCTGCTGCCTTTGTTACGC
HGF-F	GCTATCGGGGTAAAGACCTACA
HGF-R	CGTAGCGTACCTCTGGATTGC
CSPG4-F	CTTTGACCCTGACTATGTTGGC
CSPG4-R	TGCAGGCGTCCAGAGTAGA
Reference gene-GAPDH-F	CGAAGGTGGAGTCAACGGATTT
Reference gene-GAPDH-R	ATGGGTGGAATCATATTGGAAC

### 2.11. Statistical analysis

All bioinformatics analyses were conducted within R software. Differences between high- and low-risk groups were compared by Wilcoxon test. Statistical significance of risk score in different clinical subgroups was detected using ANOVA test. Finally, Student *t*-test was utilized for the expression of the characteristic genes in clinical BLCA tissue samples. If not specified, *P* < .05 was considered statistically significant.

## 3. Results

### 3.1. Identification and functional enrichment analysis of AR DEGs

**Figure 1.**
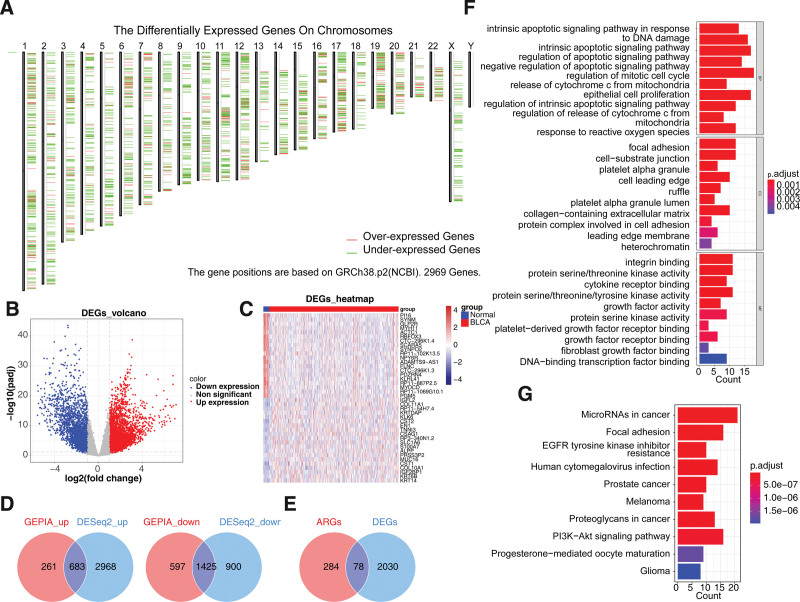
Identification and functional enrichment analysis of anoikis-related differentially expressed genes (AR DEGs) in BLCA. (A) Chromosomal localization diagram of differentially expressed genes (DEGs1) between BLCA and normal groups in TCGA-BLCA cohort (GEPIA). The screening criteria are set to |Log_2_FC| > 1 and *q*-value < 0.05. (B) Volcano plot and (C) heatmap of 5976 DEGs2 between the BLCA and normal samples (DEseq2). The screening criteria are set to |Log_2_FC| > 1 and *P <* .05. (D) Venn diagrams for 683 up-regulated and 1425 down-regulated DEGs common to GEPIA and DEseq2 tools. (E) Venn diagrams for 78 intersecting AR DEGs in BLCA. (F) The gene ontology (GO) analysis for AR DEGs. (G) The most enriched Kyoto Encyclopedia of Genes and Genomes (KEGG) terms of AR DEGs.

There were 2969 DEGs1 between BLCA and normal groups. Figure 1A illustrates the distribution of DEGs 1 on the chromosome. In total, 5976 DEGs2 were gained between BLCA and normal groups (Fig. 1B and C). Therefore, 2108 key DEGs were finally got according to the intersection of 2969 DEGs 1 and 5976 DEGs 2 (Fig. 1D). The 2108 key DEGs and 362 ARGs were intersected and 78 AR DEGs were gained (Fig. 1E). Figure 1F indicated that these AR DEGs were involved in 1234 GO items, such as intrinsic apoptotic signaling pathway in response to DNA damage, integrin binding, focal adhesion, and so on. The 99 KEGG pathways were enriched, such as microRNAs in cancer, focal adhesion, epidermal growth factor receptor (EGFR) tyrosine kinase inhibitor resistance (Fig. 1G).

### 3.2. INHBB, FASN, SATB1, CALR, HGF, and CSPG4 were identified as characteristic genes

**Figure 2.**
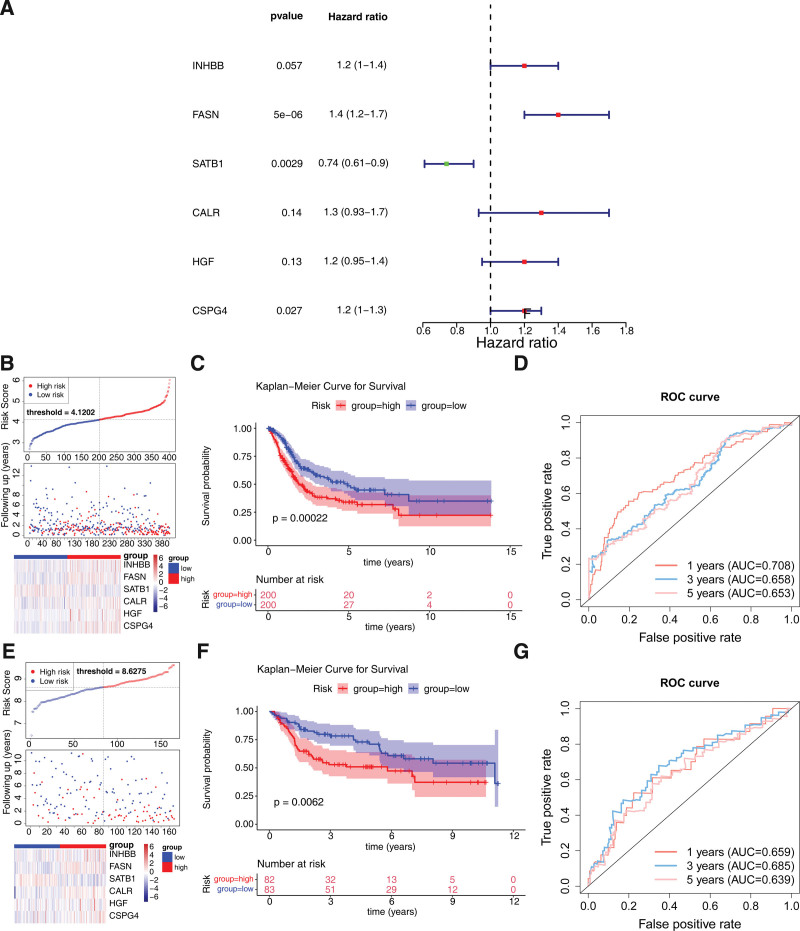
Construction of the 6-genes-based risk model in BLCA. (A) Forest plot of the multivariate COX analysis to screen 6 characteristic genes. (B) Distribution of risk scores, survival times and gene expressions of the high- and low- risk groups in the TCGA-BLCA cohorts. (C) Kaplan–Meier (K-M) survival curve of 2 risk groups in the TCGA-BLCA cohorts. (D) Receiver operating characteristic (ROC) curves for survival prediction of the TCGA-BLCA cohorts at 1, 3, and 5 yr. (E) Distribution of risk scores, survival times and gene expressions of the high- and low-risk groups in the GSE13507 cohorts. (F) K-M survival curve of 2 risk groups in the GSE13507 cohorts. (G) ROC curves for survival prediction of the GSE13507 cohorts at 1, 3, and 5 yr.

There were 21 AR DEGs that were relevant to prognosis of BLCA patients via univariate COX (Table 2). For rbsurv analysis, there were 7 AR DEGs related to prognosis of BLCA patients, containing inhibin B (INHBB), fatty acid synthase(FASN), special AT-rich sequence binding protein 1 (SATB1), calreticulin (CALR), hepatocyte growth factor (HGF), caveolin 1 (CAV1), and chondroitin sulfate proteoglycan 4 (CSPG4) (Table 3). Finally, there were 6 characteristic genes (INHBB, FASN, SATB1, CALR, HGF, CSPG4) that were selected for building a risk model through multivariate COX (Fig. 2A). Subsequently, the patients were classified into high/low-risk groups (Fig. 2B). The patients had longer survival time in low-risk group (Fig. 2C). The AUC values were 0.708 (1 year), 0.658 (3 year), and 0.653 (5 year), suggesting that the risk model had better performance (Fig. 2D). Moreover, we had carried out verification in external validation set (GSE13507). We obtained results consistent with the training set (Fig. 2E to G).

**Table 2:** Lists of 21 anoikis-related differentially expressed genes (AR DEGs) with prognostic value in BLCA that were screened by univariate Cox analysis.

Gene Symbol	HR	HR.95 L	HR.95H	*P* value
LRP1	1.352549984	1.162168725	1.574118645	9.55E-05
GLI2	1.539112632	1.190404512	1.989968679	0.001003191
IGF1	1.689416798	1.227209912	2.325705724	0.001302606
CSPG4	1.212017715	1.075824061	1.365452768	0.00156845
PDGFRA	1.275851119	1.095668797	1.485664356	0.001711839
FASN	1.271983709	1.092727145	1.480646438	0.001908279
CRYAB	1.16177644	1.053575663	1.281089288	0.0026445
SATB1	0.769655815	0.643688186	0.92027489	0.004090969
TPM1	1.183276409	1.047222884	1.337005791	0.006926404
INHBB	1.23281726	1.053602908	1.442515379	0.009015719
NTF3	0.499802452	0.289609778	0.862548539	0.012736251
CCDC178	1.860924207	1.121685038	3.087354105	0.016192485
THBS1	1.13715354	1.02351855	1.263404727	0.016723827
ITGA5	1.144921203	1.024710899	1.27923355	0.016789788
CXCL12	1.135275641	1.019141202	1.264643975	0.021204532
CAV1	1.105499144	1.01172905	1.207960133	0.026567585
ZEB1	1.216412398	1.018421578	1.452894512	0.030668102
HGF	1.227797781	1.015832194	1.483992533	0.033802587
CALR	1.372515735	1.022880175	1.841661897	0.03479251
MMP9	1.077683217	1.001917103	1.159178851	0.044277058
ILK	1.287512075	1.003640329	1.651674704	0.046750092

**Table 3:** Lists for 7 anoikis-related differentially expressed genes (AR DEGs) with prognostic value screened by rbsurv analysis.

Symbol	AIC	Selected
INHBB	937.78	1877.56*
FASN	931.74	1867.47*
SATB1	925.19	1856.39*
CALR	924.12	1856.24*
HGF	921.98	1853.96*
CAV1	921.59	1855.19*
CSPG4	919.62	1853.24*

*P < 0.05, which is statistically significant.

### 3.3. Screening of independent prognostic factors and construction of nomogram

**Figure 3.**
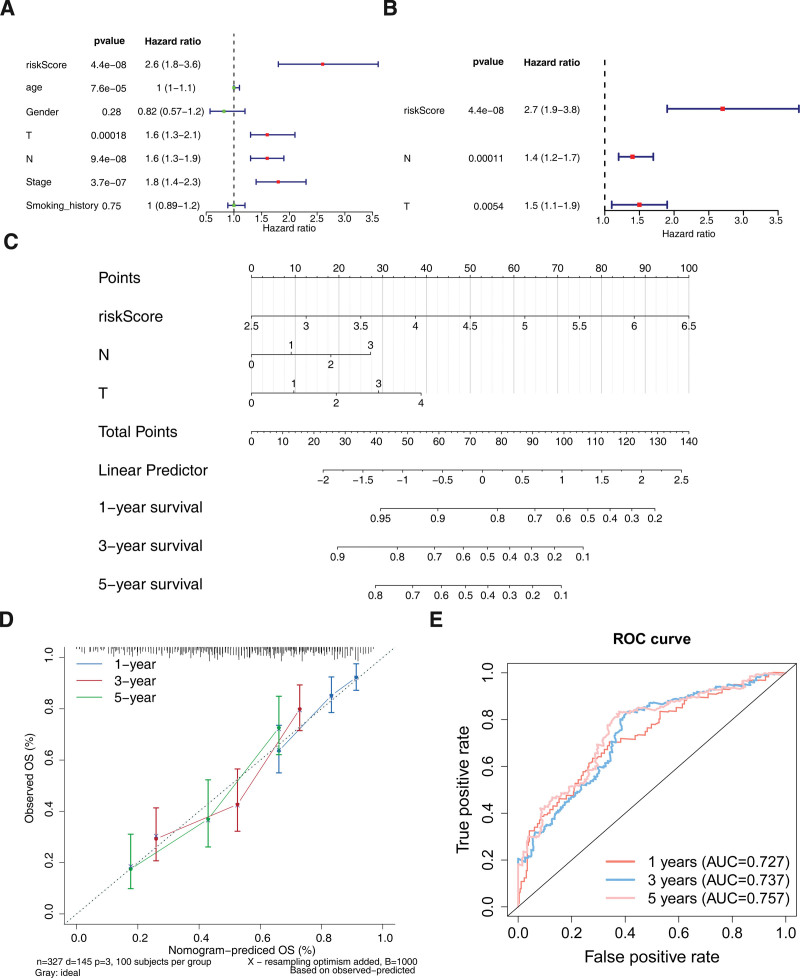
Construction of the nomogram based on risk score and other independent prognostic factors. (Aand B) Univariate and multivariate COX analysis to perform independent prognostic analysis. (C) Nomogram was constructed based on risk score, pathologic-T, and pathologic-N. (D) Calibration curve of nomogram. (E) ROC curves for predictive performance of nomogram at 1, 3, 5 yr.

There were 3 independent prognostic factors for BLCA via COX analysis, namely risk score, pathologic-T, and pathologic-N (Fig. 3A and B). As shown in Figure 3C, the nomogram contained the independent prognostic factors. The calibration curve results showed that the predicted slope was close to actual slope (Fig. 3D). The AUC values were 0.727 (1 year), 0.737 (3 year), and 0.757 (5 year) (Fig. 3E). These results suggested that the nomogram could effectively predict the risk profile of BLCA patients.

### 3.4. Analysis of risk scores and different clinical characteristics

**Figure 4.**
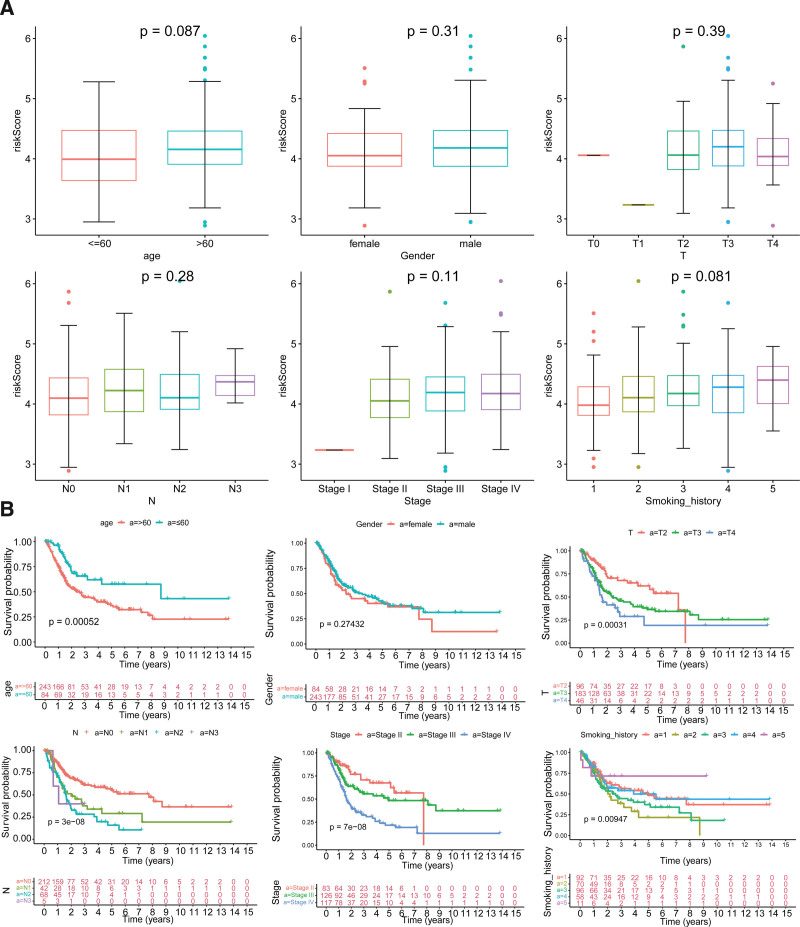
Clinical correlation analysis of the risk score and survival analysis among different clinical subgroups. (A) Boxplots for the risk score in different clinical subgroups. (B) Survival curves among different clinical subgroups.

To assess the correlation between clinical characteristics and risk scores, the differences of risk score were compared in different groups of clinical characteristics. The results demonstrated there was no marked variability in risk score among the different groups of clinical characteristics (Fig. 4A). However, the survival rate of BLCA patients was significantly differential in different groups of age, stage, smoking history, pathologic-T, and pathologic-N (Fig. 4B).

### 3.5. GO and KEGG enrichment analyses of DEGs 3

**Figure 5.**
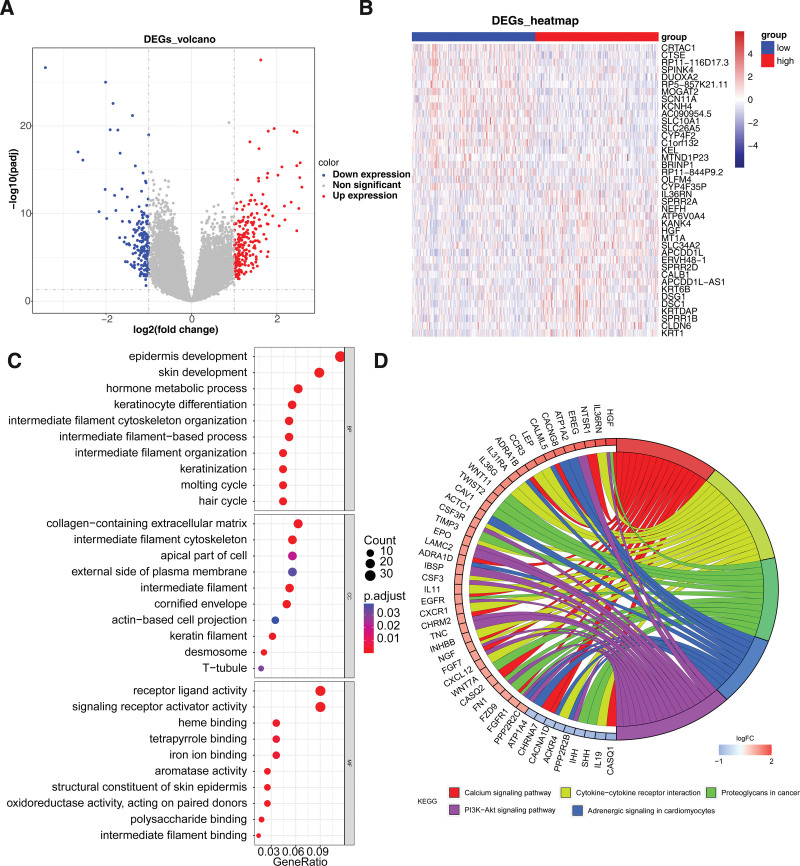
Screening and analysis of DEGs between the high- and low-risk groups. (A) Volcano plot and (B) heatmap of 397 DEGs between 2 risk groups. (C) Bubble diagram for the enriched GO terms of DEGs. (D) Circle chart for the enriched KEGG pathways of DEGs.

In total, 397 DEGs 3 (221 up-regulated and 176 down-regulated) were acquired between 2 risk groups (Fig. 5A and B). The DEGs were involved in 216 GO items and 8 KEGG pathways (Fig. 5C and D). For instance, in GO BP, the DEGs enriched in epidermis development, skin development, intermediate filament cytoskeleton organization, and so on (Fig. 5C). In GO CC, there were 15 items enriched, such as cornified envelope, intermediate filament, and intermediate filament cytoskeleton (Fig. 5C). In GO MF, the DEGs were involved in aromatase activity, receptor ligand activity, signaling receptor activator activity, etc (Fig. 5C). Of KEGG results, the DEGs enriched in calcium signaling pathway, cytokine–cytokine receptor interaction, proteoglycans in cancer, and so on (Fig. 5D).

### 3.6. A total of 56 drugs were closely associated with BLCA patients

**Figure 6.**
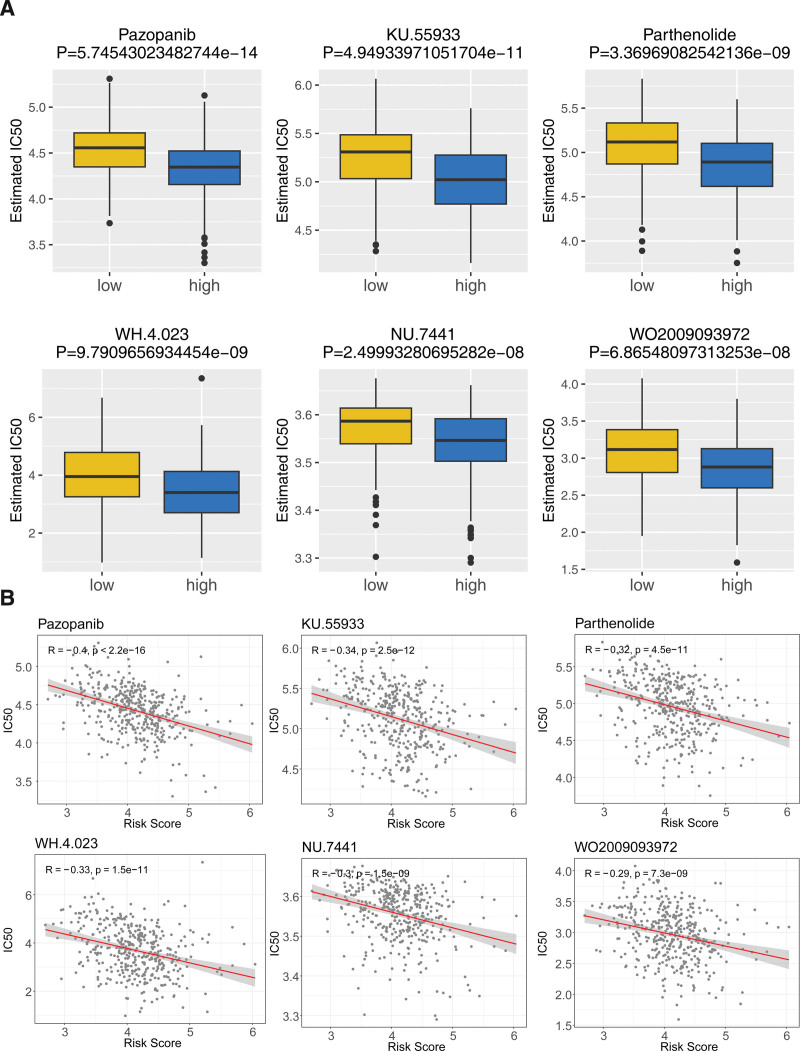
Drug sensitivity analysis of different risk groups. (A) Boxplots for the half maximal inhibitory concentration (IC_50_) of 6 differential chemotherapy drugs between the high- and low-risk groups. (B) Correlation scatter plots of the IC_50_ levels of chemotherapy drugs and risk score of BLCA individuals.

There are 56 drugs with significant differences between the 2 risk groups (Table 4), with the top 6 drugs being Pazopanib, KU.55933, Parthenolide, WH.4.023, NU.7441, and WO2009093972 (Fig. 6A). Among the 56 drugs, imatinib, docetaxel, dasatinib, cisplatin, NVP-BEZ235, and paclitaxel are closely related to bladder cancer (Table 4). The relevance between IC_50_ of differential drugs and risk score was illustrated in Table 4. The IC_50_ values of Pazopanib, KU.55933, Parthenolide, WH.4.023, NU.7441, and WO2009093972 were all negatively correlated with the risk score (Fig. 6B).

**Table 4:** Lists for 56 drugs with different half maximal inhibitory concentration (IC50) values in the high- and low-risk groups.

Drug name	*P* value	Correlation of IC50 and risk scores
Pazopanib	5.75E−14	Negative correlation
KU.55933	4.95E−11	Negative correlation
Parthenolide	3.37E−09	Negative correlation
WH.4.023	9.79E−09	Negative correlation
NU.7441	2.50E−08	Negative correlation
WO2009093972	6.87E−08	Negative correlation
PHA.665752	1.20E−07	Negative correlation
Imatinib	1.73E−07	Negative correlation
CGP.082996	2.92E−07	Negative correlation
CMK	3.03E−07	Negative correlation
TW.37	4.66E−07	Negative correlation
BX.795	8.65E−07	Negative correlation
Cyclopamine	9.67E−07	Negative correlation
Bexarotene	1.17E−06	Negative correlation
GSK269962A	1.29E−06	Negative correlation
Sunitinib	1.35E−06	Negative correlation
Z.LLNle.CHO	3.03E−06	Negative correlation
KIN001.135	4.81E−06	Negative correlation
AZD.0530	8.44E−06	Negative correlation
CGP.60474	1.24E−05	Negative correlation
AMG.706	2.09E−05	Negative correlation
Docetaxel	7.16E−05	Negative correlation
BMS.509744	8.54E−05	Negative correlation
BI.D1870	0.000114696	Negative correlation
CEP.701	0.000140433	Negative correlation
A.770041	0.000156282	Negative correlation
NVP.TAE684	0.000182619	Negative correlation
XMD8.85	0.000239516	Negative correlation
Midostaurin	0.000575211	Negative correlation
GDC0941	0.00061121	Negative correlation
PD.173074	0.001436391	Negative correlation
AZD6482	0.002136003	Negative correlation
PF.4708671	0.002657586	Negative correlation
Elesclomol	0.002855477	Negative correlation
Dasatinib	0.003248836	Negative correlation
AZD7762	0.003352314	Negative correlation
AUY922	0.004292721	Negative correlation
Embelin	0.004919625	Negative correlation
Cisplatin	0.005910855	Negative correlation
NVP.BEZ235	0.006013221	Negative correlation
Cytarabine	0.006207506	Negative correlation
GW843682X	0.006548983	Negative correlation
GNF.2	0.008241089	Negative correlation
GDC.0449	0.00839807	Negative correlation
PF.02341066	0.010025174	Negative correlation
AICAR	0.011549968	Negative correlation
Axitinib	0.011867298	Negative correlation
Paclitaxel	0.012496701	Negative correlation
Obatoclax.Mesylate	0.016553151	Negative correlation
S.Trityl.L.cysteine	0.021687607	Negative correlation
RO.3306	0.021867643	Negative correlation
CHIR.99021	0.02330617	Negative correlation
JNK.Inhibitor.VIII	0.036105307	Negative correlation
MG.132	0.043753393	Negative correlation
BMS.708163	0.048344004	Negative correlation
FTI.277	0.049395089	Negative correlation

### 3.7. Expression validation of characteristic genes in mRNA level

**Figure 7.**
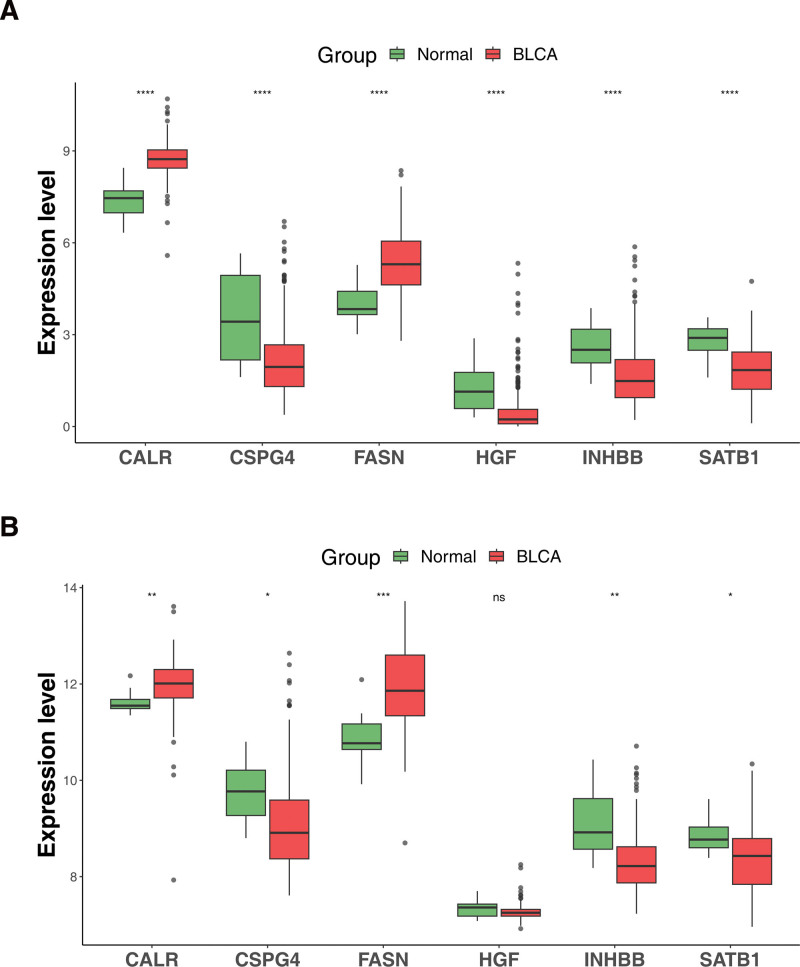
Boxplots for the expression levels of 6 characteristic genes in different BLCA-related datasets. (A) TCGA-BLCA cohort, (B) GSE13507. *P < .05, **P < .01, ***P < .001, ****P < .0001.

Through the visualized data, we found that the expression trends of the 6 characteristic genes were identical in the TCGA-BLCA cohort and GSE13507 (Fig. 7A and B). The expression of all characteristic genes between BLCA and normal groups was considerably different in TCGA-BLCA cohort (Fig. 7A). However, the expression level of HGF between BLCA and normal groups was not significant in GSE13507 (Fig. 7B). Both TCGA-BLCA cohort and GSE13507, CALR and FASN were up-regulated in BLCA groups, while the expression of CSPG4, HGF, INHBB, and SATB1 was opposite.

**Figure 8.**
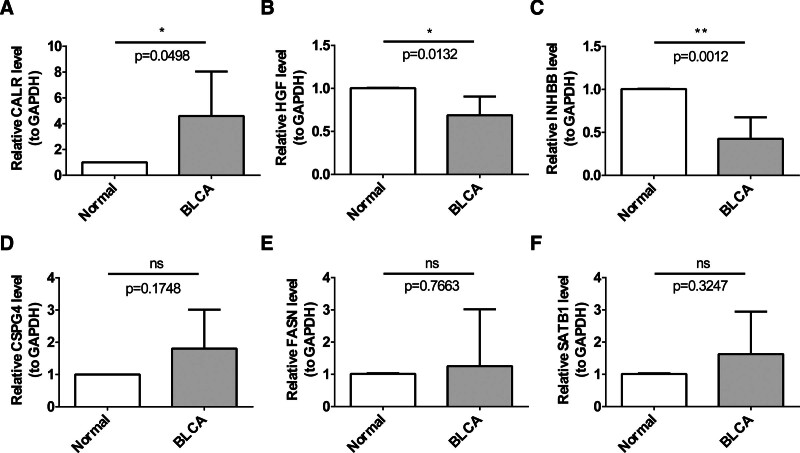
The expressions of 6 characteristic genes in clinical BLCA and normal tissue samples were identified by real time quantitative-polymerase chain reaction (RT-qPCR). (A) CALR, (B) HGF, (C) INHBB, (D) CSPG4, (E) FASN, and (F) SATB1. *P < .05.

The expression of CALR, HGF, and INHBB between BLCA and normal groups was different apparently by RT-qPCR results (Fig. 8A to C). Moreover, the expression trends of CALR, HGF, and INHBB were accordant with the previous results obtained from public databases. However, the expression of CSPG4, FASN, and SATB1 between BLCA and normal groups was not significantly different, while was higher in BLCA group (Fig. 8D to F). The expression trend of CSPG4 and SATB1 was the contrary to that of the public datasets, which may be due to heterogeneity in the samples, as well as differences in the experimental design.^[16]^

## 4. Discussion

BLCA is a highly malignant tumor with poor prognosis and limited treatment options, often exhibits high recurrence rates.^[17]^ Increasing evidence suggests that ARGs may serve as effective biomarkers for predicting the prognosis of a variety of tumors. For example, researchers have found that 14-3-3σ protein promotes hepatocellular carcinoma (HCC) cell survival and proliferation by inhibiting the degradation of EGFR, which in turn activates the EGFR-dependent ERK1/2 signaling pathway.^[18]^ Research suggests that 5 genes (BAK1, SPP1, BSG, PBK, and DAP3) are risk factors for anoikis in HCC, and there is a strong correlation between infiltrating immune cells and these 5 prognostic genes.^[19]^ The genes CYBB, CPA3, and SFRP2, which are related to anoikis and immunity, have been identified as key genes affecting the prognostic prediction in colorectal cancer.^[20]^ Yet, the relationship between ARGs and the prognosis of BLCA remains unclear.

In our study, we identified characteristic genes (CALR, FASN, CSPG4, HGF, INHBB, and SATB1), which were associated with anoikis in BLCA patients. Based on these genes, we constructed a risk model that effectively predicted the prognosis of BLCA patients.^[21]^

CALR, situated within the Endoplasmic Reticulum (ER), is involved in various cellular processes. CALR surface expression on BLCA cells facilitates the recognition and clearance by phagocytes, thereby initiating an immune response against cancer.^[22]^ Mutations impairing CALR function can disrupt the homeostatic balance in healthy cells, contributing to the development of tumors. Additionally, CALR plays a significant role in resistance to anoikis through its intracellular functions as a calcium ion buffer and its involvement in integrin-mediated cell adhesion.^[23]^ Calcium-activated chloride channel A4 (CLCA4) could mediate the migration and incursion of BLCA cells by regulating EMT and PI3K/AKT activation.^[24]^ Reduced expression of CALR inhibits the adhesion of BLCA cells to type I collagen and suppresses the metastasis of BLCA by regulating the phosphorylation of focal adhesion proteins.^[25]^ In BLCA, an increase in CALR expression correlates with unfavorable disease-specific survival and a reduction in Overall Survival (OS).^[26]^ Enhanced CALR levels in both tumor samples and urine from individuals with BLCA may be utilized as prospective indicators.^[27]^ FASN can synthesize the lipids needed by BLCA cells, providing the energy and material basis for tumor cells to grow, and thus was effective in promoting the growth, invasion, and metastasis of cancer cells. FASN is significantly correlated with the immune microenvironment, immune cell infiltration, and immune function of BLCA, and it affects the infiltration of CD4 + T cells, neutrophils, and dendritic cells compared to patients with high expression of FASN, patients with low expression of FASN are more sensitive to anti-PD-1 and anti-CTLA-4 therapies.^[28]^ By downregulating the FASN protein, BLCA cells become sensitive to vincristine again.^[29]^ Researchers have demonstrated that the downregulation of FASN exerts an inhibitory effect on gastric cancer cells with AR by suppressing the p-ERK1/2/Bcl-xL signaling pathway.^[30]^ In osteosarcoma cells undergoing AR, the inhibition of FASN can reduce lung metastasis, and a similar decrease in the levels of p-ERK1/2 and Bcl-xL is also observed.^[31]^ CSPG4 functions as a cell surface proteoglycan and promotes oncogenesis.^[32]^ In BLCA, elevated expression of CSPG4 indicates a poor prognosis.^[33]^ Researchers have found that BLCA tissues that became resistant after treatment with cisplatin and gemcitabine showed a significant increase in CSPG4 expression.^[34]^ CSPG4 modulates interactions with extracellular matrix receptors, its decomposition, and assembly processes, thereby influencing pathways linked to epithelial–mesenchymal transition (EMT), which collectively propel tumor progression. It is particularly noteworthy that downregulation of CSPG4 expression markedly suppresses PD-L1 expression.^[35]^ HGF is a protein that is crucial for cell growth, proliferation, and differentiation. HGF, secreted by mesenchymal cells, facilitates interactions between malignant cells and fibroblasts associated with cancer, fostering a microenvironment conducive to tumorigenesis and tumor progression. It stimulates inflammatory responses, cell migration, blood vessel formation, and the invasive spread characteristic of metastasis.^[36]^ Patients treated with HGF inhibitor capmatinib showed a significant improvement in progression-free survival compared to those who underwent standard chemotherapy.^[37]^ Studies have shown that elevated HGF expression is associated with decreased progression-free survival and overall survival in patients with BLCA.^[38]^ HGF binding to its receptor MET triggers the dimerization and autophosphorylation of MET, activating downstream signaling pathways, such as the activation of the PI3K/AKT pathway that promotes cell survival and inhibits anoikis.^[39]^ Epithelial/Mesenchymal (E/M) hybrid cancer cells possess stable AR, invasive capacity, and tumorigenic potential, with HGF being the primary driver that induces cancer cells to enter the E/M hybrid state.^[40]^ Inhibin B(INHBB) is a glycoprotein that belongs to the transforming growth factor-β (TGF-β) family and is composed of a common α-subunit and a β-subunit, forming a heterodimer. INHBB reduces the phosphorylation of Smad2/3 to inhibit EMT, indicating that INHBB may suppress the AR and migratory capacity of nasopharyngeal carcinoma (NPC) cells through the TGF-β/Smads signaling pathway. The decrease in INHBB expression is also associated with the overexpression of p53, which may further affect the invasiveness and metastatic potential of NPC cells.^[41]^ INHBB acts as a tumor suppressor in nasopharyngeal carcinoma cells but acts a tumor promoter in gastric and colorectal cancers.^[42,43]^ Currently, there is no research to verify the expression of INHBB in BLCA. In our study, we found that INHBB is under-expressed in BLCA, suggesting that it may be a protective factor. SATB1 is a protein that binds to DNA at nuclear matrix attachment regions. It can influence the EMT in BLCA cells, which is a key step in tumor invasion and metastasis. Additionally, SATB1 can regulate various signaling pathways and target genes, such as ERK, AKT, and p53, thereby affecting tumor cell growth, apoptosis, metastasis, and drug resistance.^[44]^ Overexpression of SATB1 in BLCA tissues correlates with higher tumor stages and lymph node metastasis.^[45]^ The Hepatitis B Virus X protein (HBx) stimulates SATB1 expression by triggering the ERK and p38 MAPK signaling pathways, which helps prevent anoikis and facilitates the spread of liver cancer cells.^[46]^

The analysis of dataset revealed up-regulated expression of CALR and FASN, and down-regulated expression of CSPG4, HGF, INHBB, and SATB1. This contrasts with the reported expression patterns in the relevant literature, where INHBB expression in BLCA is uncertain, and CALR, FASN, CSPG4, HGF, and SATB1 are highly expressed. The discrepancy between the 2 trends could be attributed to variations in sample sources or overall experimental design. For the first time, we have demonstrated that INHBB is under-expressed in BLCA. The role of these characteristic genes in BLCA contributes to a deeper understanding of the pathogenesis and clinical manifestations of the disease, offering new targets and strategies for the diagnosis and treatment of BLCA.

Our study revealed that compared to the low-risk group, some drugs in the high-risk group have lower IC_50_ values and are more sensitive to the drugs. Utilizing this characteristic can guide the formulation of personalized chemotherapy plans for high-risk BLCA patients, extend survival periods, and delay the resistance to chemotherapy drugs. Drug sensitivity analysis plays an essential role in personalized treatment plans. Therefore, we performed the analysis to identify which treatments may be effective against BLCA. We found 6 drugs – imatinib, docetaxel, dasatinib, cisplatin, NVP-BEZ235, and paclitaxel – associated with the treatment of BLCA.

Imatinib enhances the sensitivity of tumor cells to radiation therapy by suppressing the RAD51 protein involved in the Homologous Recombination pathway. Given that muscle-invasive bladder cancer (MIBC) cells have impairments in the Non-Homologous End-Joining mechanism, a combined approach of radiotherapy and Imatinib can exploit this deficiency to augment therapeutic outcomes while potentially reducing damage to normal tissues.^[47]^ Furthermore, Imatinib has the capability to suppress tumor growth by inhibiting platelet-derived growth factor receptor (PDGFR) and other tyrosine kinase receptors, thereby preventing the formation of blood vessels that are essential for tumor development.^[48]^ Docetaxel (DTX) inhibits the polymerization of microtubules in cancer cells, thereby preventing their proliferation and metastasis. When DTX is used to treat high-risk nonmuscle-invasive bladder cancer (NMIBC) patients after standard bacillus Calmette–Guérin (BCG) therapy failure, the initial complete response rate reaches 60%, with a 3-year recurrence-free survival (RFS) rate of 25%.^[49]^ Researchers observed that, among individuals with high-grade NMIBC, the combination of DTX and gemcitabine demonstrated superior outcomes in terms of high-grade RFS and lower treatment discontinuation rates in comparison to the BCG therapy, with a noted difference of 2.9% versus 9.2%, respectively.^[50]^ Dasatinib, a type of orally administered inhibitor for SRC family kinases, exhibits efficacy against urothelial tumor cells. Neoadjuvant dasatinib was shown to be safe and feasible for patients with muscle-invasive urothelial carcinoma of the bladder in a prospective multicenter phase II trial.^[51]^ In cases of MIBC with EGFR gene amplification and loss of phosphatase and tensin homolog, dasatinib demonstrates antitumor activity. It suppresses the SRC and PI3K/AKT/mTOR pathways by decreasing the phosphorylation of SRC at Tyr416 and AKT at Ser473, leading to antiproliferative and pro-apoptotic effects.^[52]^ Cisplatin hinders tumor cell proliferation and triggers cell death by creating intrastrand DNA crosslinks and forming DNA-protein adducts. When combined with 5-fluorouracil for MIBC, cisplatin enhances the sensitization effect of radiotherapy, achieving a complete response rate of up to 88%.^[53]^ Studies have shown that the knockout of the heterogeneous nuclear ribonucleoprotein U gene escalates the sensitivity of BLCA cells to cisplatin, promoting cell cycle arrest and apoptosis induced by the drug, and consequently reducing cellular migration.^[54]^ Systematic review and meta-analysis suggest that cisplatin-based adjuvant chemotherapy improves RFS, locoregional RFS, and metastasis-free survival in patients with muscle-invasive BLCA.^[55]^ SATB1 depletion in 5637 and T24 BLCA cells reduces cell proliferation while upregulating cisplatin-induced apoptosis.^[56]^ NVP-BEZ235, a compound that targets both phosphatidylinositol 3-kinase (PI3K) and mammalian target of rapamycin complex 1/2 (mTORC1/2). Matsushima et al showed a decreasing trend in bladder weight and levels of pAKT, pS6, and p4EBP1 in the NVP-BEZ235-treated group compared with the control group, suggesting that NVP-BEZ235 exerts a significant antitumor effect by inhibiting the PI3K/AKT/mTOR pathway.^[57]^ The combination of NVP-BEZ235 with cisplatin demonstrates significant synergistic antitumor effects in cisplatin-resistant BLCA cells. The co-treatment reduces the IC_50_ values of NVP-BEZ235 and cisplatin by 5.6-fold and 3.6-fold, respectively.^[58]^ Based on this, it is speculated that NVP-BEZ235 could be attempted in the treatment of cisplatin-resistant BLCA. Paclitaxel targets the cellular spindle and causes the arrest of eukaryotic cells in the G2/M phase, ultimately inducing apoptosis.^[59]^ Paclitaxel can interact with microRNAs to modulate the mTOR signaling pathway, thereby enhancing its antitumor efficacy. For instance, paclitaxel upregulates the expression of miR-143, which post-transcriptionally suppresses the expression of AKT.^[60]^ The paclitaxel-combination therapy, including carboplatin, exhibits a response rate of 14% to 65% in advanced urothelial carcinoma and is particularly suitable for patients with renal insufficiency.^[61]^ In our study, we observed that the IC_50_ values of 6 drugs – imatinib, docetaxel, dasatinib, cisplatin, NVP-BEZ235, and paclitaxel – were relatively low in the context of high-risk BLCA, suggesting a heightened sensitivity of the cancer to these agents. This insight is beneficial for informing clinical pharmaceutical decision-making and may lead to enhanced treatment efficacy.

Our study screened characteristic genes to predict the prognosis of BLCA patients. However, our study primarily involves bioinformatics analysis, supplemented by RT-qPCR validation of the expression levels of ARGs. Therefore, the mechanism of ARGs which were associated with the prognosis of BLCA patients involved in the development of BLCA needs further study.

## 5. Conclusion

In summary, this study employed a series of bioinformatics methods including differential expression analysis, enrichment analysis, univariate Cox analysis, multivariate Cox analysis, prognostic model construction, and chemotherapy drug sensitivity analysis, to screen for characteristic genes related to anoikis in BLCA patients. Based on characteristic genes, a risk model was constructed that accurately predicts survival rates of BLCA. Furthermore, significant differences in sensitive chemotherapy drugs were identified between high- and low-risk groups. This study has important clinical significance for the diagnosis and treatment of BLCA patients.

## Acknowledgments

This study was supported by the High-Level Medical Expert Training Program of Guangxi “139” Plan Funding (G202002016) and the Research Program of the Health Commission of Guangxi Zhuang Autonomous Region (Z-A20220572).

## Author contributions

**Resources:** Fu Huang, Xuyong Sun.

**Writing – original draft:** Fu Huang.

**Supervision:** Liquan Zhou, Xiaoming Wang, Fuzhi Long, Haipeng Huang.

**Formal analysis:** Junjie Sun, Fuzhi Long, Haipeng Huang.

**Investigation:** Junjie Sun, Jiange Zhang.

**Project administration:** Xihua Ma.

**Methodology:** Yongfeng Pei, Qiuwen Zhang, Yanqing Yu, Guining He, Lirong Zhu, Xiaoming Wang.

**Software:** Haibin Li, Jiange Zhang.

**Visualization:** Haibin Li.

**Writing – review & editing:** Xuyong Sun.
